# Editorial: A new perspective of human CD8+ T cells with respect to their biology, protective and pathogenic role in anti-viral and tumor responses

**DOI:** 10.3389/fimmu.2023.1321224

**Published:** 2023-10-31

**Authors:** Soumya Panigrahi, Powel Kalinski, Aki Hoji

**Affiliations:** ^1^ Division of Infectious Diseases and HIV Medicine, Case Western Reserve University School of Medicine, Cleveland, OH, United States; ^2^ Department of Immunology, Roswell Park Comprehensive Cancer Center, Buffalo, NY, United States; ^3^ Department of Pediatrics, Indiana University-Purdue University School of Medicine, Indianapolis, IN, United States

**Keywords:** immunology, immunotherapy, COVID-19, cancer, malaria

Focusing in to the areas of recent interests, the research presented in this section opens up a Research Topic of studies in immunology and immunotherapy, together that make a substantial impact within the scientific community and beyond. These original works collectively underscore the crucial role of the immune system and T-cell immune responses in the context of COVID-19, cancer, and malaria, offering fresh perspectives on CD8+ T cell biology and their roles in both protection and pathogenesis. The investigation into aging-related changes in CD8+ T cells during COVID-19 infection highlights the need for tailored interventions for older individuals, while the study on tumor-infiltrating lymphocytes presents promising avenues for personalized cancer treatments. In the realm of gastric cancer, the research on CD226 sheds light on potential prognostic indicators and the complex interactions within the tumor microenvironment. Finally, the innovative approach to malaria vaccine development displays a path forward in the fight against this global health crisis.

The COVID-19 pandemic has brought light to the critical role of immune responses in determining disease severity, particularly among the elderly population (1, 2). In the study titled “*Distinct SARS-CoV-2 specific NLRP3 and IL‐1β responses in T cells of aging patients during acute COVID-19 infection*” by Mahalingam et al., a comprehensive investigation into the impact of aging on the immune response of CD4+ and CD8+ T cells in individuals infected with SARS-CoV-2 is presented. By characterizing T cells from individuals aged 61 and above (i.e. higher risk) to those aged below 60, the research clearly illuminates significant aging-related alterations in T cells, contributing to an amplified immune response in older individuals during acute COVID-19 infection, irrespective of disease severity. Notably, T cells from individuals older than 60’s exhibited a diminished capacity to produce IFN-γ and IL-1β when stimulated with SARS-CoV-2 peptides *in vitro*. Additionally, these T cells displayed elevated PD-1 expression, with expected reduction in IFN-γ/PD-1 ratios upon stimulation, implying a potential compromise in their functional efficacy. The most importantly, the study showed reduced expression of IL-1β and NLRP3, pivotal components in the antiviral immune response, in T lymphocytes of older individuals, while monocytes remained unaffected. These findings provide crucial insights into the novel mechanisms underlying T cell dysregulation in COVID-19 responses within the ageing population, shedding light on avenues for refining therapeutic interventions and vaccine strategies tailored to this vulnerable population (3, 4).

In the realm of cancer immunotherapy, the study conducted by Aran et al., titled “*Analysis of tumor infiltrating CD4+ and CD8+ CDR3 sequences reveals shared features putatively associated to the anti-tumor immune response*,” investigates the potential of tumor-infiltrating lymphocytes (TILs) as natural resources for antitumor responses. Focusing on engineered transgenic T cell receptors (TCRs) to bolster cancer cell recognition, the study delves into the TCR repertoires of CD4+ and CD8+ TILs in breast cancer (BC) (5, 6). Despite limitations in biopsy extraction yielding limited TILs, the study successfully sequenced TCR from individual CD4+ and CD8+ TILs and compared them to those TCRs from circulating CD4+ and CD8+ in healthy donors. The findings uncovered vital distinctions in TCRs between T cell subsets, potentially influenced by tumor antigens. Notably, CD4+ TILs exhibited reduced TCR diversity yet shared conserved TCR motifs, hinting at the identification of antigen-specific tumor-reactive cells. Furthermore, CD4+ and CD8+ TILs displayed disparities in TCR repertoires, suggesting potential ramifications for their antigen recognition capabilities. This study thus lays the foundation for personalized treatment strategies utilizing class I and class II restricted TCRs, honing in on those critical tumor antigens that are associated with improved clinical outcomes.

Turning attention to gastric cancer (GC), a prominent global cause of cancer-related mortality, and the study titled “*CD226 Identifies Functional CD8+ T Cells in the Tumor Microenvironment and Predicts a Favorable Outcome for Human Gastric Cancer*” by Huang et al. illuminates promising avenues for personalized treatment approaches. By assessing molecular biomarkers such as MSI, PD-L1, and HER2, the study offers potential insights into refining immunotherapy and targeted therapy outcomes (7). Of particular interest is the investigation into immune cell subsets within GC tissue, which holds promise for predicting responses to immunotherapy and patient prognosis. The study underscores the significance of CD226 (DNAM1), widely expressed on various immune cells, in enhancing CD8+ T cell-mediated antitumor responses. TIGIT and CD96, established inhibitory checkpoint receptors in the tumor microenvironment, provide counterbalance. The study unveils elevated peripheral TIGIT expression in CD8+ T cells of GC patients and reduced peripheral CD226+CD8+ T cell levels compared to healthy controls. Furthermore, heightened CD226 expression in cancer tissues correlates with improved patient outcomes. This study underscores the potential of CD226 as a prognostic indicator in GC, offering valuable insights into the intricate interplay between CD226, infiltrating CD8+ T cells CD8+ T cells, and tumor cells within the GC tumor microenvironment. The findings hold implications for advancing personalized treatment strategies in the fight against gastric cancer.

Malaria persists as a global health crisis, claiming over half a million lives annually. Addressing this urgent need, the study by Padula et al., titled “*Induction of Antigen-Specific Intrahepatic CD8+ T Cell Responses by a Secreted Heat Shock Protein-Based gp96-Ig-PfCA Malaria Vaccine*,” introduces a pioneering approach to malaria vaccine development (8). Focusing on disrupting malaria liver-stage parasite development, the study presents a novel platform centered on a secreted form of the heat shock protein, gp96-immunoglobulin (gp96-Ig), to elicit antigen-specific memory CD8+ T cell responses against malaria. By employing engineered HEK-293 cells expressing gp96-Ig and established Plasmodium falciparum vaccine candidate antigens, the research achieved robust responses in murine and non-human primate models. Notably, the study strategically engineered properties of gp96-Ig to assess its efficacy in generating Plasmodium antigen-specific, liver-resident, memory CD8+ T cells. These findings illuminated a substantial portion of CSP and AMA1-specific CD8+ T cells with conventional tissue-resident memory T cell phenotypic markers, CD69 and CXCR3, alongside their secretion of IL-2, vital for sustaining long-term memory responses effective in the liver. Significantly, the unique dual functionality of gp96-Ig, acting as an adjuvant and antigen carrier, amplifies its technical advantage in the development of innovative vaccine approaches, not only for malaria but also for other infectious diseases. This study overall marks a significant stride towards inducing critical liver-homing, antigen-specific CD8+ T cells, pivotal for shielding against Plasmodium liver-stage infections, underscoring the imperative for further advancements in secreted gp96-Ig technology within the broader landscape of malaria vaccine research (9).

The insights provided are highly relevant to various critical areas such as the COVID-19 pandemic, public health, personalized cancer treatments, and guiding the worldwide battle against malaria. These findings promise to not only enhance scientific comprehension but also hold the potential to benefit patients through advancements in future therapeutic interventions and the availability of improved vaccine strategies. Essentially, this section underscores the pivotal role of immunology research in tackling urgent global health challenges, rendering it exceptionally influential within the scientific community and in broader endeavors to enhance healthcare outcomes worldwide.

## Author contributions

SP: Conceptualization, Writing – original draft, Writing – review & editing. PK: Supervision, Writing – review & editing. AH: Writing – review & editing.
